# Evaluation of the concordance of sialometry and salivary glands scintigraphy in dry mouth patients

**DOI:** 10.1016/S1808-8694(15)30043-4

**Published:** 2015-10-19

**Authors:** Bianca Maria Liquidato, Rita de Cássia Soler, Ivo Bussoloti Filho

**Affiliations:** a PhD in otolaryngology from the F.C.M. Santa Casa de São Paulo - Prof. Instructor of the Morphology Department - F.C.M. Santa Casa de São Paulo; b PhD in otolaryngology from the F.C.M. Santa Casa de São Paulo – Prof. Instructor of the Hospital Nossa Sra. de Lourdes; c PhD in otolaryngology from the UNIFESP – Assistant Professor of the Otolaryngology department - Faculdade de Ciências Médicas da Santa Casa de São Paulo. Otolaryngology Department of the Medical Sciences School of the Santa Casa de São Paulo

**Keywords:** xerostomia, salivary glands, saliva, diagnosis

## Abstract

**Introduction:**

Many diagnostic tests are used to evaluate dry mouth patients, especially the ones with Sjögren's Syndrome, to whom these tests are part of classification criteria for scientific studies.

**Aim:**

Thus, the concordance between results of sialometry and salivary glands scintigraphy was evaluated; if positive, it would enable the choice of one or the other for diagnosis.

**Patients and Method:**

Seventy-two dry mouth patients were divided into non-Sjögren's Syndrome group, primary Sjögren's Syndrome group and secondary Sjögren's Syndrome group. The concordance among sialometry and scintigraphy results was evaluated by Kappa test.

**Results:**

It was observed that their concordance was equal or near to zero.

**Conclusion:**

It is not possible to make a choice between these tests and both should be performed.

## INTRODUCTION

The assessment of dry mouth complaining patients involves a number of questions, both to confirm the complaint as well as to try and define one etiology^1^, ^2^.

The oral cavity exam may bring important information about the oral mucosa situation and the possible complications of salivary secretion reduction^3^, ^4^.

Besides the clinical evaluation, a number of tests should be performed in order to determine salivary flow alterations^1^, ^5^. Sjögren's syndrome is among the various causes of dry mouth^1^, ^2^, and once there is no test to ascertain its diagnosis, there are classification criteria used to include patients in the scientific studies^6^, ^7^.

The criteria used to classify both primary and secondary Sjögren's syndrome, has been broadly discussed in the international literature.

Such discussion involves not only the set of criteria to be used, in other words, those published by many groups, but also the ones from Copenhagen, San Diego, the Greek, the Japanese and the ones from the European Community Study Group/American-European Consensus, but also the tests that should be carried out^6^, ^7^, ^8^, ^9^. At any rate, most sets of criteria provide us with options as to the tests to be carried out in order to fulfill a certain item of a set of criteria.

The European Community Study Group tried to validate a number of tests that had their specificity and sensitivity tested in this way^10^, ^11^.

Thus, the ones with the best performance were included as part of this set of criteria.

In order to evaluate the involvement of the greater salivary glands, they chose salivary gland scintigraphy; parotid gland sialography and the non-stimulated salivary flow sialometry^9^, ^10^, ^11^, ^12^, ^13^, ^14^.

Among them, both the sialometry^15^, ^16^, ^17^ and the scintigraphy^18^, ^19^ assess the gland function, not only its image.

When altered, any of these tests fulfill the criterion^13^, ^14^. Sialometry is a simple, low cost test, and of easy execution as long as the technique is standardized^16^.

Scintigraphy bears advantages that include the saliva quantitative analysis throughout the evaluation of radiopharmaceutical build up and salivary escape, which may correctly reflect the functional alterations and bring information about obstructive clogging of the salivary gland.

It may also detect early stages of salivary gland involvement^18^, ^19^, ^20^. Because sialometry and scintigraphy are easily executed tests and provide information about the glandular function, often times they are preferred over parotid sialography, which is also a more invasive type of test.

For these reasons and also because of the great current cost concern, let alone the major technological innovations, we tried to assess if there would be an correspondence between the salivary gland sialometry and the scintigraphy so that, if present, we could chose one of the two tests.

## MATERIALS AND METHODS

All dry mouth patients who came to the Otolaryngology Department of the Santa Casa de São Paulo, were then referred to the stomatology outpatient ward of the institution.

**Chart 1:**
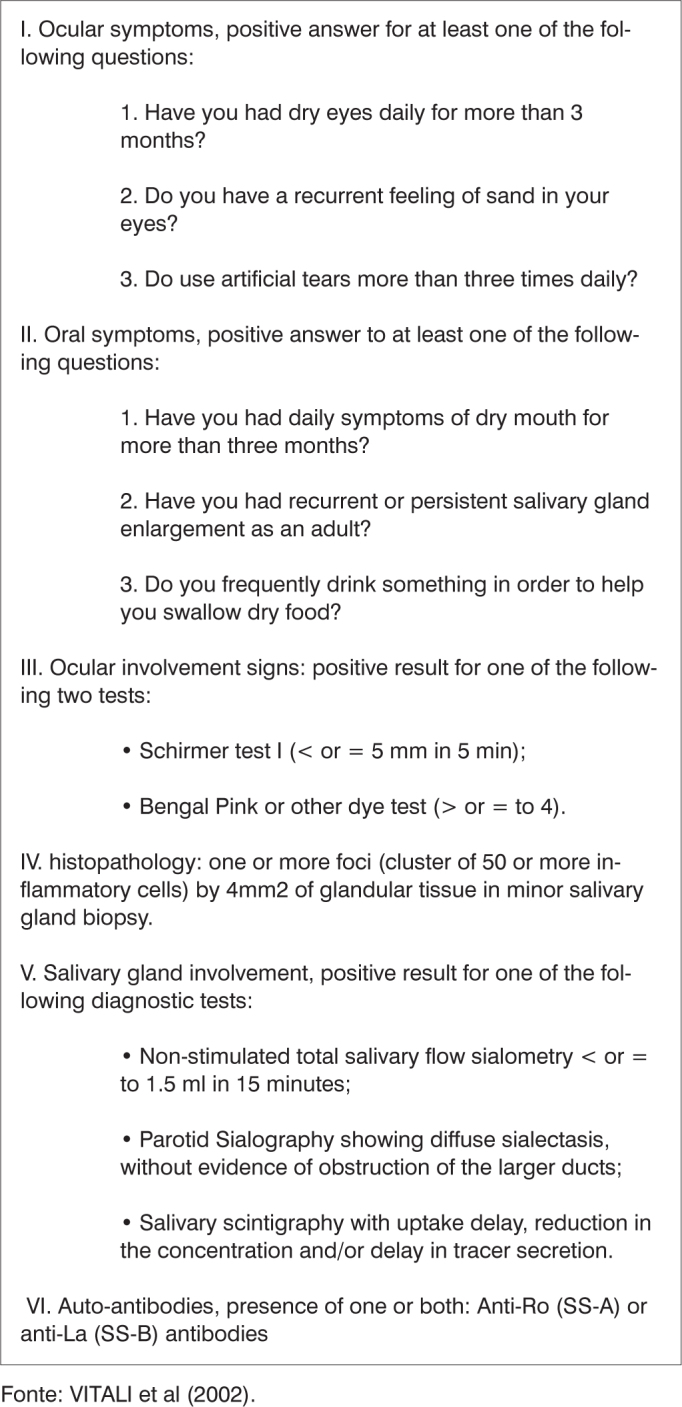
Sjögren syndrome classification criteria

From January 1997 until December 2003, 72 patients with this complaint were clinically assessed and underwent diagnostic evaluation and classification, based on the criteria established at the American-European Consensus^14^ (Chart 1).

Fonte: VITALI et al (2002).

Based on such criteria, these patients were divided in 2 groups: one group that did not have Sjögren syndrome (NSS) and a group with Sjögren syndrome (SS).

The Sjögren syndrome group was further divided in 2 subgroups: with primary Sjögren syndrome (SSp) and secondary Sjögren syndrome (SSsec).

In order to classify the patients with primary Sjögren syndrome it was necessary to have 4 of the 6 items, items IV (histopathology) or VI (autoantibodies) were mandatory.

As far as the secondary Sjögren syndrome patients classification is concerned, this was done with the presence of either item I or II, plus any other among items III, IV and V (Chart 1). Exclusion criteria for Sjögren syndrome classification were previous head and neck radiotherapy; pre-existent lymphoma; sarcoidosis; graft versus host disease; anti-cholinergic drug use.

There was also one Wegener granulomatosis patient that was excluded.

Criterion V, assessment of major salivary gland involvement encompasses, as previously mentioned, an altered salivary glands sialometry or scintigraphy, or parotid sialography.

Non stimulated sialometry was carried out through the saliva collection technique using two cotton balls which had been previously weighed together with the universal collecting 80ml jar, in a digital scale.

Patients were advised to swallow all the saliva they had in their oral cavity and the cotton balls were then placed on the mouth floor, close to the gingival border, where they remained for 2 minutes.

After this time span the whole set was again weighed.

The weight difference was directly changed from g/min into ml/minute and was considered as altered with values below 0.1 ml/minute.

The salivary gland scintigraphy was carried out assessing sodium pertechnetate technetium uptake in the 15 mCi dose and its subsequent clearance after salivary stimulation with 8 ml of lemon juice on the tongue dorsum, 20 minutes after the radiopharmaceutical injection.

Scintigraphies with delayed uptake; reduced concentration and/or delay in the tracer secretion were considered altered. The statistical methodology used was the Kappa measure, which is an correspondence measure where the 0 value indicates no correspondence and the 1 value indicates total correspondence.

Kappa was then calculated for the NSS, SSp, and SSsec groups and for all the patients.

In order to check whether the correspondence was reasonable, a statistical test was carried out to assess Kappa significance.

The significance index used was 5%. The Institution Ethics Committee approved such research.

## RESULTS

The Kappa values found were different from zero for all cases and for the NSS group, however for the SSp and SSsec groups, such values were equal to zero (Table 1). In groups where the correspondence index was different from zero, the values were very low.

**Table 1:** Kappa results calculated for the different groups

	Kappa	Standard error	p
All cases	0,22	0,09	0,018
NSS Group	0,36	0,11	0,003
SSp Group	-0,11	0,10	0,506
SSsec Group	-0,23	0,21	0,236

NSS = non-Sjögren syndrome; SSp = primary Sjögren syndrome; SSsec = secondary Sjögren syndrome

## DISCUSSION

According to the current attempt to reduce costs, if it were possible to choose between one of the tests, the choice would be the sialometry, because it is a low cost test that does not expose the patient to radiation.

The very fact of the Kappa index that assesses the correspondence of the test results be equal to zero or very low, indicates that we may not choose one of the tests only.

This low or absent correspondence may be due to the fact that sialometry assesses glandular function only, while scintigraphy also assesses radioisotope uptake by the gland, that may be influenced by circulatory alterations.

At any rate, both tests may be subject to variations related to fluctuations in the quantity of saliva produced.

This fact alone confirms the need to use diagnostic criteria that involve more then one test to assess the involvement of the greater salivary glands.

## CONCLUSION

Both sialometry and scintigraphy should be carried out in order to assess the involvement of salivary glands in patients complaining of dry mouth, specially in Sjögren syndrome patients, since one can not replace the other because of a lack of correspondence among their results.
